# Development and validation of an activatable PET radiotracer reporting extracellular myeloperoxidase activity for the detection of unstable atherosclerotic plaque

**DOI:** 10.1038/s44303-026-00156-9

**Published:** 2026-04-07

**Authors:** George P. Keeling, Xiaoying Wang, Weiyu Chen, Nadia Chaher, Ling Gao, Marcelo E. Andia, Sergey Tumanov, Piotr Golda, Mohammed M. Chowdhury, Prakash Saha, Lefteris Livieratos, James Nadel, Roland Stocker, Alkystis Phinikaridou

**Affiliations:** 1 School of Biomedical Engineering and Imaging Sciences, Res. Dept. of Cardiovascular Imaging, King’s College London, London, UK; 2 Heart Research Institute, Newtown, NSW Australia; 3 Faculty of Medicine and Health, The University of Sydney, NSW 2006 Sydney, Australia; 4 Guangdong Key Laboratory of Vascular Diseases, Guangzhou Institute of Cardiovascular Disease, The Second Affiliated Hospital, Guangzhou Medical University, Guangzhou, China; 5 School of Medicine, Pontificia Universidad Católica de Chile, Santiago, Chile; 6 London Metallomics Facility, King’s College London, London, UK; 7 Department of Vascular Surgery, University of Cambridge, Cambridge, UK; 8 Nuclear Medicine Department, Guy’s & St Thomas’ Hospitals NHS Foundation Trust, London, UK; 9 St Vincent’s Hospital, Sydney, NSW Australia; 10 King’s BHF Centre of Research Excellence, London, UK

**Keywords:** Biochemistry, Biomarkers, Cardiology, Diseases

## Abstract

Extracellular arterial activity of the pro-inflammatory enzyme myeloperoxidase (MPO) destabilizes atherosclerotic plaque and associates with future atherothrombosis. To facilitate first-in-human studies using extracellular MPO activity as a molecular imaging target to identify high-risk atherosclerotic plaque, we describe [^68^Ga]Ga-IEMA, a NODAGA-based positron emission tomography (PET) radiotracer that provides an index for extracellular MPO activity. Synthesis of [^68^Ga]Ga-IEMA was achieved in five steps and with high radiolabelling efficiency. [^68^Ga]Ga-IEMA self-oligomerized and bound to proteins upon exposure to enzymatically active MPO, did not cross-cell membranes and was stable in human serum in vitro, while [^68^Ga]Ga-IEMA had favorable blood kinetics and stability in circulation in vivo. [^68^Ga]Ga-IEMA PET imaging in a mouse model of plaque instability revealed enhanced signal in unstable compared with stable plaque and plaque-free arteries. These data indicate that [^68^Ga]Ga-IEMA is a promising translational candidate for the non-invasive identification of high-risk atherosclerotic plaques and the evaluation of therapies targeting arterial inflammation.

## Introduction

Atherosclerosis remains a primary cause of disability and premature death worldwide^1^. Arterial inflammation is a key driver in the formation and destabilization of atherosclerotic plaques with an unstable plaque phenotype that drives most clinical events, including myocardial infarction and stroke^2–5^. Imaging of arterial inflammation in atherosclerosis and tracking the anti-inflammatory effects of therapeutics has been reported using magnetic resonance imaging (MRI)^6–8^, positron emission tomography/computed tomography (PET/CT)^9–13^, nanoparticles^14^, and PET/MRI^15^. Nevertheless, non-invasive imaging of inflammation remains challenging because inflammation has two facets: a “pro-inflammatory” response that causes damage to the host and formation of unstable plaques, and a “reparative” response, which can be beneficial, promoting repair and giving rise to stable plaques.

Inflammation is often assessed by imaging macrophages, and the most used approach, i.e., PET/CT imaging using [^18^F]-fluorodeoxyglucose ([^18^F]-FDG), does not differentiate pro-inflammatory from anti-inflammatory/reparative macrophages. Additionally, when examined histologically, there is a weak correlation between the extent of [^18^F]-FDG uptake by macrophages and their expression of pro-inflammatory markers. [^68^Ga]Ga-DOTATATE, which targets the somatostatin receptor SSTR2, has been reported to offer greater specificity for pro-inflammatory macrophages and better power to discriminate high-risk versus low-risk coronary lesions than [^18^F]-FDG^16^. The claimed specificity of SSTR2 for pro-inflammatory macrophages in atherosclerotic lesions^16^ is based on in vitro experiments and the co-expression of SSTR2 and CD68. Importantly, however, CD68 is a pan-macrophage marker^17^ and can be expressed by both pro- and anti-inflammatory macrophages^18^, and imaging probes that specifically differentiate between disease-promoting and disease-reparative inflammation are not used clinically. To improve risk stratification and direct personalized therapy, a molecular target specific for a pro-inflammatory activity that directly causes plaque destabilization and contributes to clinical events in patients at elevated risk is needed.

The pro-inflammatory enzyme myeloperoxidase (MPO) represents such a potential biomarker^19^. MPO is expressed by neutrophils and pro-inflammatory macrophages but not by anti-inflammatory macrophages^20–22^, and it catalyzes the formation of hypochlorous acid and other reactive oxygen and nitrogen species^23^ that contribute to the killing of intracellular microbes. MPO can be released into the extracellular space via degranulation, apoptosis, extravascular migration, or via neutrophil extracellular traps (NETs)^23^. Extracellular MPO activity destabilizes plaque via matrix degradation and cap thinning by activating matrix metalloproteinases and inactivating their inhibitors, and it contributes to plaque erosion and increased propensity for thrombus formation by causing endothelial cell apoptosis^24^.

There is substantive evidence for a direct role of MPO activity in the formation of unstable, high-risk atherosclerotic plaque. Firstly, pre-clinical studies in a mouse model of plaque instability have demonstrated that MPO activity is significantly higher in unstable compared with stable plaque; genetic deletion of *MPO* prevents the formation of unstable plaque; and pharmacological inhibition of MPO stabilizes pre-existing unstable plaque by increasing cap thickness without changing the content of plaque inflammatory cells^25,26^. Secondly, increased plaque MPO activity predicts plaque rupture/erosion and ensuing thrombosis in a rabbit model of atherothrombosis^27^.

There is also strong evidence for a role of increased MPO activity in high-risk human atherosclerosis. Unstable human coronary and carotid plaques have increased MPO activity compared with stable plaques, and increased MPO activity in carotid plaque associates with adverse clinical outcomes in stroke patients^28^. These recent findings are consistent with earlier studies reporting the presence of MPO activity in both advanced human atheroma^24^ and culprit coronary plaque responsible for fatal acute myocardial infarcts^29^.

Molecular imaging of MPO activity has been achieved using MPO-activable probes such as the MRI probes MPO-Gd^30^ and heMAMP^31^ that report extracellular MPO activity, and the [^18^F]MAPP PET radiotracer^32,33^ that detects both intra- and extracellular MPO activity. MPO-specific oxidation of the phenol group of the 5-hydroxytryptamide moiety forms probe-derived radicals and gives rise to probe oligomerization and probe–protein adduct formation. This results in higher retention on the reacted probe and hence higher molecular imaging signal at sites of increased MPO activity. In pre-clinical models, in vivo molecular MRI of arterial MPO activity detects plaque inflammation^34^, differentially identifies unstable from stable atherosclerotic plaques^25,34^, and prospectively identifies plaques that cause atherothrombosis^27^.

Despite strong data establishing extracellular MPO activity as an imaging target to identify high-risk atherosclerotic plaque, clinical translation of this approach is yet to be achieved. Here, we set out to develop and validate an activatable PET radiotracer that reports on extracellular MPO activity, analogous to the MRI probes but different to [^18^F]MAPP, to significantly lower the barrier for clinical translation. PET is a quantitative modality that provides higher spatial resolution than other nuclear imaging techniques in humans, and it can be coupled with computed tomography coronary angiography (CTCA) to permit the assessment of plaque composition, stenotic grade, and inflammatory activity. Development of a PET radiotracer for direct and quantitative assessment of extracellular MPO activity could enable the detection of inflamed and unstable atherosclerotic plaques non-invasively, identifying patients at elevated risk of cardiovascular events.

## Results

### Synthesis and radiolabelling of IEMA

**Fig. 1:**
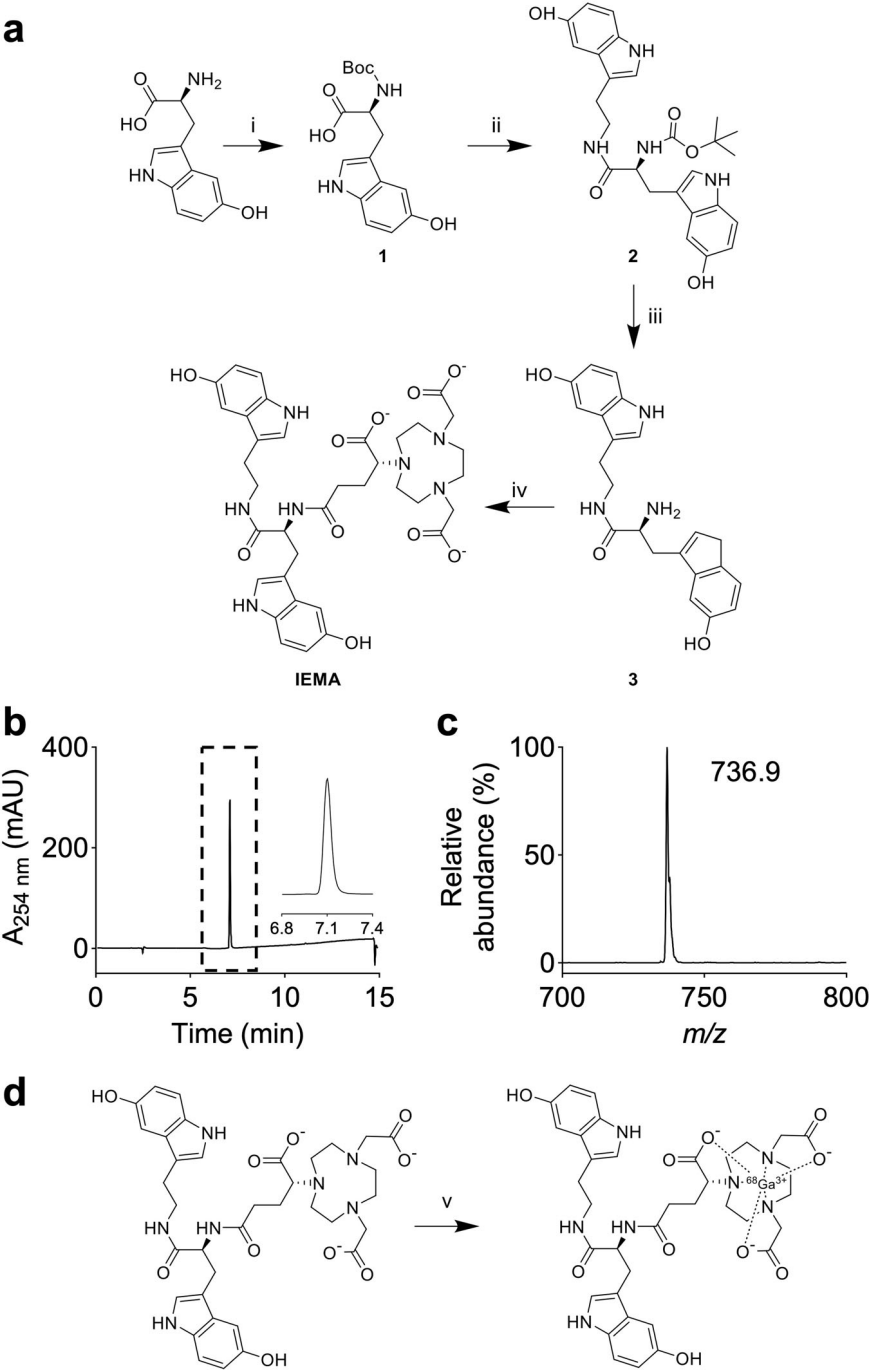
Probe synthesis. **a** Scheme of the synthesis of IEMA. Conditions and reagents used in steps: (i) Boc anhydride, K_2_CO_3_, tetrahydrofuran, H_2_O, 2 h, room temperature (RT); (ii) 5-hydroxy-L-tryptophan hydrochloride (0.8 equiv.), 1-[bis(dimethylamino)methylene]-1H-1,2,3-triazolo[4,5-b]pyridinium3-oxid hexafluorophosphate (2 equiv.), diisopropylethylamine (DIPEA), dimethylformamide (DMF), 24 h, RT, 31% yield after 2 steps; (iii) trifluoroacetic acid/dichloromethane, 24 h, RT, 16% yield; (iv) (R)-NODAGA-NHS (2.5 equiv.), DIPEA, dry DMF, N_2_, 4 h, RT, 18% yield. **b** Reversed-phase high-performance liquid chromatography (HPLC) chromatogram of the purified product (IEMA) after step iv with the eluate monitored at 254 nm. Inset shows zoom of peak eluting at 7.1 min (HPLC Method 1). **c** Mass spectrum of compound eluting at 7.1 min in (**b**) as determined by liquid chromatography mass spectrometry. **d** Radiosynthesis of [^68^Ga]Ga-IEMA. Conditions and reagents used were: 28 µM IEMA, 1.2 M sodium formate buffer, [^68^Ga]GaCl_3_ in 0.1 M HCl, pH 3.5, 10 min, 50 °C.

The synthetic route towards IEMA, i.e., the immediate precursor of [^68^Ga]Ga-IEMA, is shown in Fig. 1a. Synthesis began with Boc-protection of 5-hydroxy-L-tryptophan to give intermediate 1, which underwent an amide coupling reaction with serotonin using HATU as the coupling reagent to yield intermediate 2. Subsequent deprotection using trifluoroacetic acid/dichloromethane produced intermediate 3, containing an amine group available for subsequent coupling with NODAGA-NHS ester under basic conditions to give IEMA.

The purity and chemical identity of IEMA were verified by reversed-phase HPLC (Method 1; Fig. 1b), LC/MS (Fig. 1c), and ^1^H NMR. Radiolabelling of IEMA was performed in a single complexation step without pre-processing of the ^68^Ge/^68^Ga generator eluate or purification steps. The labeling was performed at pH 3.5, using a formate-based buffer, and the radiochemical purity of isolated [^68^Ga]Ga-IEMA ranged from 95 to 100% as assessed by ITLC (*n* = 44 separate preparations). The radiosynthesis (Fig. 1d) could be performed within 11 min, allowing for activity yields up to 89% from the start of the radiosynthesis, and molar activities recorded in the range 11.9–29.8 MBq nmol^–1^.

### In vitro characterization of [^68^Ga]Ga-IEMA

**Fig. 2:**
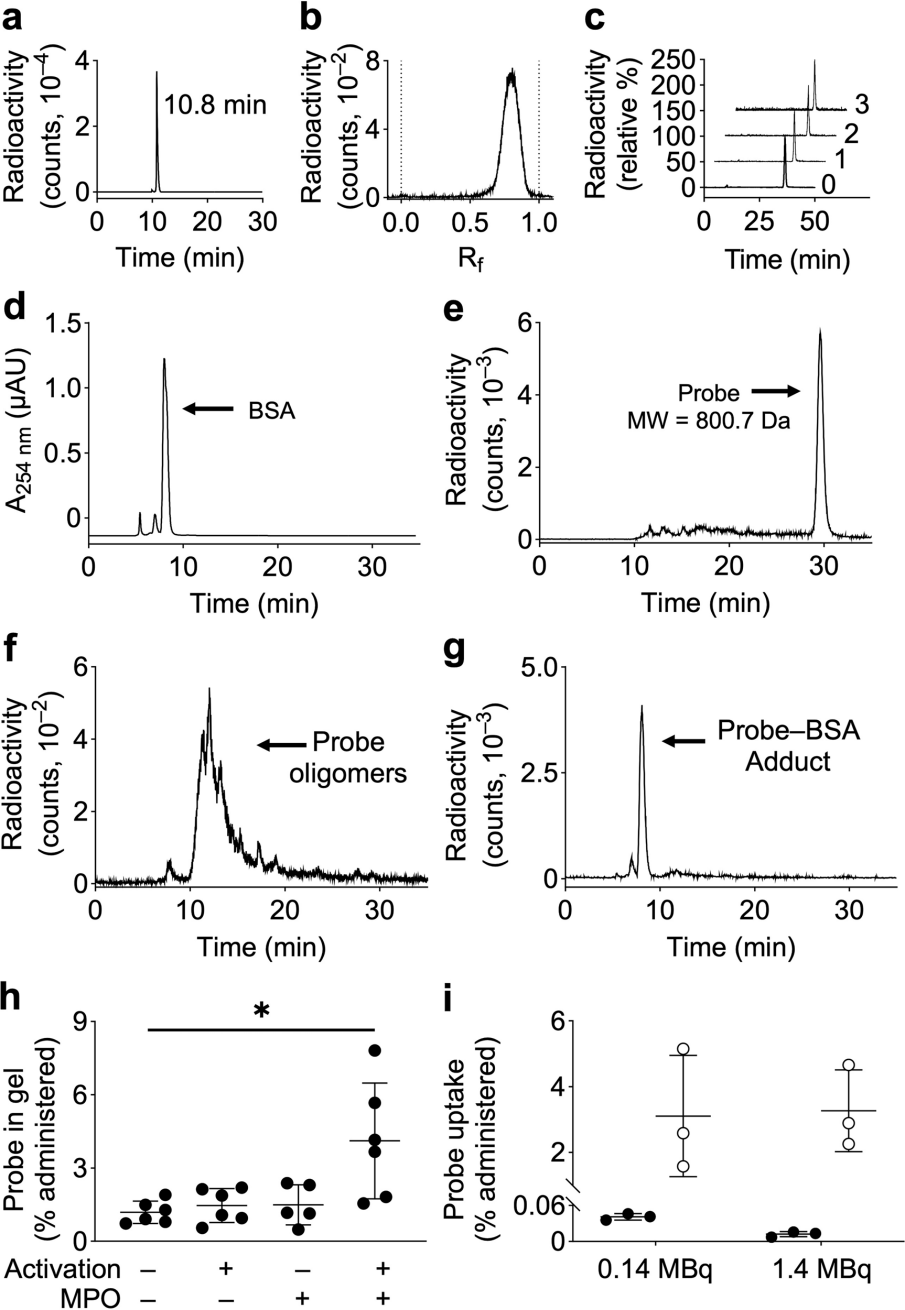
In vitro characterization of [^68^Ga]Ga-IEMA. **a** Reversed-phase radio-HPLC chromatogram of [^68^Ga]Ga-IEMA shows a single major peak accounting for >98% of total radioactivity. **b** Instant thin layer chromatography of [^68^Ga]Ga-IEMA shows >99% of signal at *R*_f_ = 0.85. **c** Size exclusion HPLC chromatograms of [^68^Ga]Ga-IEMA added to human blood serum and incubated at 37 °C for 3 h, with 97.4 ± 0.5, 96.6 ± 0.2, and 95.9 ± 1.8% area remaining in the primary peak after 1, 2, and 3 h, respectively (mean ± SD of three independent experiments). **d**–**g** Binding of [^68^Ga]Ga-IEMA to protein during incubation of bovine serum albumin (BSA) with myeloperoxidase (MPO), glucose, and glucose oxidase (GOX) at 37 °C for 1 h as assessed by size exclusion radio-HPLC. Representative chromatograms of **d** BSA, **e** [^68^Ga]Ga-IEMA, and MPO-activated [^68^Ga]Ga-IEMA in (**f**) the absence and **g** presence of BSA (**g**). Results of a single experiment performed in triplicate are shown. **h** Binding of [^68^Ga]Ga-IEMA to MPO in Hybrid Gel. Hybrid Gel without (–) and with MPO (+) was incubated for 1 h at 37 °C with PBS (control) or glucose and GOX. Results of 5–6 independent experiments are shown. *P < 0.05 (Kruskal–Wallis). **i** Validation of lack of cellular uptake of [^68^Ga]Ga-IEMA (filled circles). Human umbilical endothelial cells were incubated for 60 min at 37 °C with 0.14 or 1.4 MBq [^68^Ga]Ga-IEMA or neutralized non-chelated (free) ^68^Ga (open circles) before the cells were centrifuged and the cell pellets and supernates examined for radioactivity. Results of a single experiment performed in triplicate are shown.

The newly synthesized radiotracer was characterized in vitro in several ways. First, reversed-phase radio-HPLC of [^68^Ga]Ga-IEMA revealed a single major peak accounting for >98% of total radioactivity (Fig. 2a), and instant thin layer chromatography showed >95% of the radioactivity signal at *R*_f_ = 0.85 (Fig. 2b). Second, the lipophilicity of [^68^Ga]Ga-IEMA was assessed by determining its LogP and LogD_7.4_ values using its partitioning into octanol in the presence of water and PBS, respectively. Three separate experiments, performed with 3 separate batches of freshly prepared [^68^Ga]Ga-IEMA, gave LogP-values ranging from −2.23 to −2.30 and LogD_7.4_-values ranging from −2.23 to −2.25 (LogD_7.4_). These results indicate that [^68^Ga]Ga-IEMA was predominantly in the aqueous phase and, according to the trends encompassed by Lipinski’s Rule of 5 and relevant literature^35,36^, unlikely to pass membrane bilayers. Third, freshly prepared [^68^Ga]Ga-IEMA was incubated under air and at 37 °C with freshly thawed, commercial human serum in PBS. To prevent oxidation of the probe under air, the radical scavenger, sodium ascorbate, was added. Probe stability was assessed by size exclusion radio-HPLC using PBS as mobile phase at 1 mL/min and determined as the percentage radioactivity signal detected in the major peak eluting at 36 min. The percentage radioactivity recovered was 97.4 ± 0.5, 96.6 ± 0.2, and 95.9 ± 1.8% after 60, 120, and 180 min, respectively (*n* = 3 independent experiments). These results indicate excellent stability of [^68^Ga]Ga-IEMA in human serum (Fig. 2c). In addition, the stability of ^nat^Ga-IEMA was assessed in a formulation solution suitable for in vivo use (i.e., 5% ethanol/95% saline). Following storage at either room temperature (19–26 °C) for 6 h or under refrigerated conditions (2–6 °C) for 24 h, resulted in ≥98% recovery of the probe, as determined by LC-MS/MS analysis (data not shown).

### Reaction of [^68^Ga]Ga-IEMA with active MPO

The reaction of [^68^Ga]Ga-IEMA with active MPO was assessed first in solution and then in gels. Freshly prepared [^68^Ga]Ga-IEMA was incubated in PBS with various combinations of MPO and BSA in the absence and presence of hydrogen peroxide generated by glucose/GOX. Reaction mixtures were analyzed by size exclusion radio-HPLC using PBS as the mobile phase. Sodium ascorbate was not added to any part of these reactions or the mobile phase. The chromatograms of BSA and [^68^Ga]Ga-IEMA alone are shown in Fig. 2d, e, respectively, with the radiotracer eluting at 30 min. Figure 2e shows the presence of peaks eluting before 30 min, indicative of larger molecular species, likely representing oligomers of [^68^Ga]Ga-IEMA. These accounted for 27% of the radiotracer as assessed by radioactivity. In the presence of active MPO, >98% of the radioactivity eluted as putative oligomers (Fig. 2f). In the presence of [^68^Ga]Ga-IEMA and BSA (at concentrations comparable to that of albumin in human blood plasma), 85% of the radioactivity was observed to elute at the same time as BSA whilst the remaining 15% appeared as oligomeric [^68^Ga]Ga-IEMA (Fig. 2d–g).

We then tested the binding of [^68^Ga]Ga-IEMA to MPO in hybrid gels. Gels prepared with MPO, the MPO-activating combination of glucose/GOX, neither or both were incubated in PBS with [^68^Ga]Ga-IEMA. No significant difference was seen between the amount of radioactivity associated with the gel in incubations that lacked MPO or glucose/GOX (1.2 ± 0.4% activity bound) and gels that only contained MPO (1.5 ± 0.6%) or glucose/GOX (1.5 ± 0.7%). However, there was significantly higher binding of [^68^Ga]Ga-IEMA to the gel containing both MPO and glucose/GOX (4.1 ± 2.2%) (Fig. 2h).

Finally, we tested the cellular uptake of [^68^Ga]Ga-IEMA, using HUVEC as model cells. Cells incubated with either a low activity (0.14 MBq) or high activity (1.4 MBq) showed negligible uptake of [^68^Ga]Ga-IEMA (0.04 ± 0% and 0.01 ± 0% bound activity, respectively). By contrast, incubation of the cells with the same activities of free gallium-68 had a measured bound fraction of 3.1 ± 1.5 and 3.3 ± 1.0%, respectively (Fig. 2i). These results support previous findings that [^68^Ga]Ga-NODAGA-based radiopharmaceuticals do not readily enter cells^35,36^. In the case of [^68^Ga]Ga-IEMA, these results are also consistent with its low LogP and LogD_7.4_ values (see above) and provide direct evidence for the utility of [^68^Ga]Ga-IEMA as an index of extracellular MPO activity. Overall, in vitro characterization showed that [^68^Ga]Ga-IEMA is moderately hydrophilic, does not cross cell membranes, and is stable in human serum. Enzymatic activation by MPO leads to probe oligomerization and covalent protein binding.

### In vivo characterization of [^68^Ga]Ga-IEMA

**Fig. 3:**
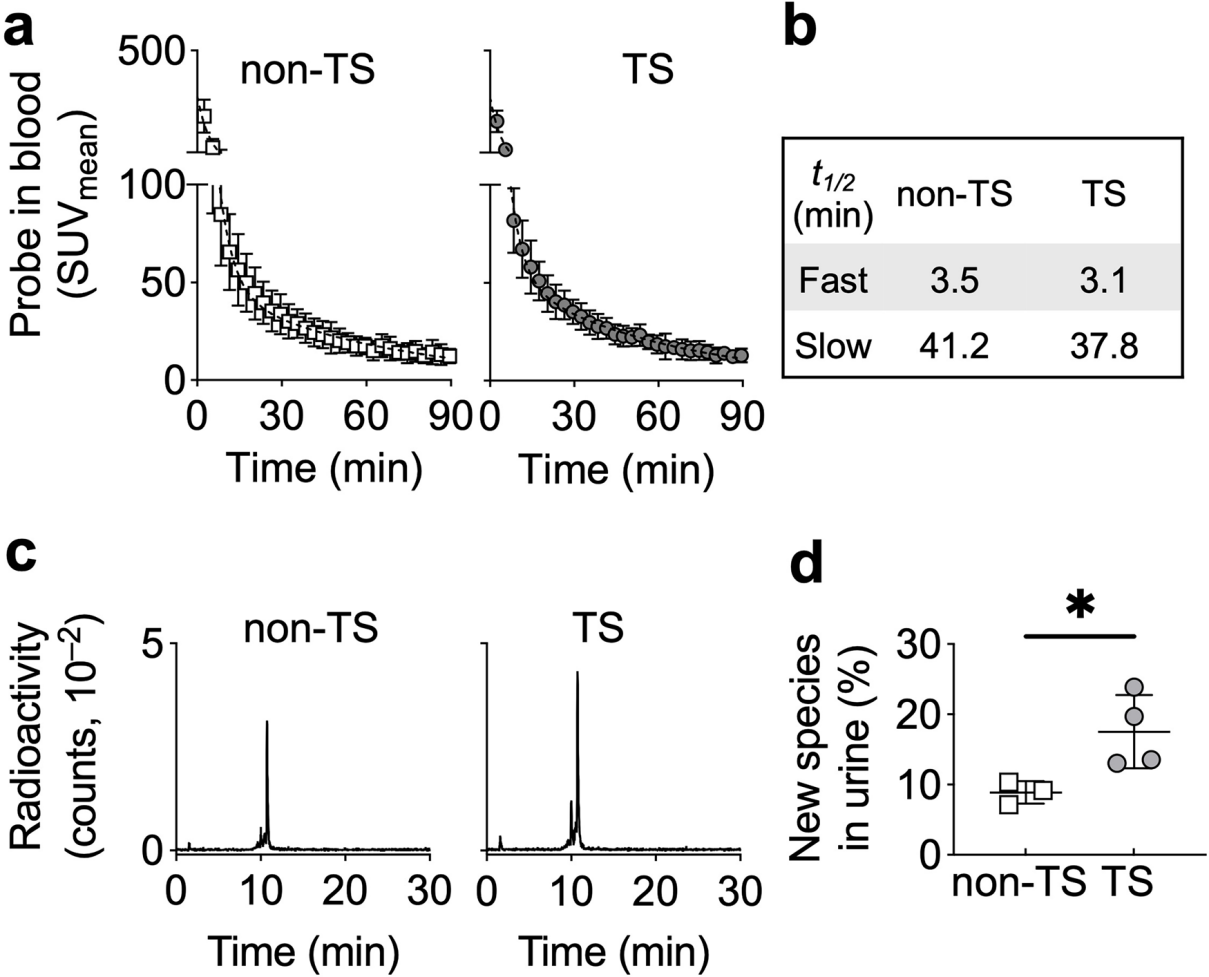
In vivo characterization of [^68^Ga]Ga-IEMA. **a**, **b** Blood kinetics of [^68^Ga]Ga-IEMA up to 90 min after intravenous administration of the radiotracer (102.6 ± 1.5 µL, 8.2 ± 3.8 MBq) into Apoe^–/–^ mice without (non-TS Apoe^–/–^, *n* = 6) and with Tandem Stenosis (TS) surgery (TS Apoe^–/–^, *n* = 8) as measured by in vivo positron emission tomography (PET) imaging of the left ventricle. Image analysis using an exponential two-phase decay model shows that [^68^Ga]Ga-IEMA was eliminated from the blood in a biexponential manner typical of radiotracers with extracellular distribution and renal clearance, as indicated in (**b**). **c** Reversed-phase HPLC of urine directly after collection from non-TS Apoe^–/–^ (*n* = 3) and TS Apoe^–/–^ (*n* = 4) mice 90 min after administration of [^68^Ga]Ga-IEMA (104.8 ± 3.8 µL, 11.4 ± 2.6 MBq) showing intact radiotracer at 10.8 min and the formation of a new compound eluting at 10.0 min. Representative chromatograms for urine obtained from non-TS Apoe^–/–^ and TS Apoe^–/–^ mice are shown. **d** Percentage of total signal (peaks at 10.8 and 10.0 min) present in the peak at 10.0 min. *P < 0.05 (Mann–Whitney test).

Next, we investigated the in vivo blood kinetics and ex vivo tissue biodistribution of [^68^Ga]Ga-IEMA by in vivo PET imaging of the left ventricle after intravenous administration of the radiotracer into control non-TS *Apoe*^–/–^ and TS *Apoe*^–/–^ mice, i.e., animals that did not and did undergo TS surgery, respectively. Dynamic images were acquired up to 90 min post-injection, and images were reconstructed every 3 min. Data were fitted to a two-phase decay model, and the fast and slow half-lives calculated. Both non-TS *Apoe*^–/–^ and TS *Apoe*^–/–^ mice showed comparable fast (3.5 and 3.1 min) and slow half-lives (41.2 and 37.8 min), respectively (Fig. 3a, b). Videos of the in vivo biodistribution of the radiotracer in non-TS *Apoe*^–/–^ and TS *Apoe*^–/–^ mice revealed predominant renal clearance, in conjunction with some hepatobiliary excretion (Movie S1). Ex vivo gamma counting of radioactivity from tissues collected from non-TS *Apoe*^–/–^ and TS *Apoe*^–/–^ mice 90 min after administration of [^68^Ga]Ga-IEMA showed excretion of the radiotracer mainly in the urine and low retention in the gallbladder and small intestines (Table 1). The results obtained indicate that 90 min after administration, only ~0.2% of the administered [^68^Ga]Ga-IEMA remained in blood. Dosimetry calculations showed an estimated effective dose of 3.2 and 3.9 mSv per 200 MBq in male and female mice, respectively.

**Table 1:** Ex vivo biodistribution of [^68^Ga]Ga-IEMA 90 min after intravenous administration to *Apoe*^–/–^ mice.

Tissue	Radiotracer uptake (SUV)	
	non-TS	TS
	(*n* = 5)	(*n* = 5)
Urine	115 ± 53.4	99.9 ± 50.1
Gallbladder	24.5 ± 22.4	24.5 ± 19.7
Small intestine	11.80 ± 0.83	11.2 ± 2.10
Kidneys	1.20 ± 0.14	1.28 ± 0.28
Liver	1.02 ± 0.11	1.10 ± 0.17
Thoracic aorta	0.51 ± 0.34	0.57 ± 0.21
Tail	0.83 ± 0.37	0.67 ± 0.12
Skin & fur	0.45 ± 0.07	0.48 ± 0.11
Stomach	0.39 ± 0.33	0.53 ± 0.50
Blood	0.34 ± 0.10	0.33 ± 0.08
Large intestine	0.18 ± 0.07	0.27 ± 0.08
Lungs	0.36 ± 0.10	0.24 ± 0.08
Spleen	0.28 ± 0.11	0.23 ± 0.06
Tibia	0.12 ± 0.02	0.15 ± 0.04
Heart	0.085 ± 0.031	0.079 ± 0.015
Thymus	0.074 ± 0.029	0.088 ± 0.047
Muscle	0.052 ± 0.015	0.055 ± 0.016
Brain	0.011 ± 0.004	0.011 ± 0.006

Finally, to assess the extent of in vivo metabolism of [^68^Ga]Ga-IEMA, urine was collected directly from the bladder at termination of the experiment (90 min after radiotracer administration) and analyzed by reversed-phase radio-HPLC without further sample processing. The primary peak, eluting at 10.8 min, corresponded to that of [^68^Ga]Ga-IEMA, whilst a secondary peak which eluted at 10.0 min was also observed (Fig. 3c). In non-TS *Apoe*^–/–^ mice, this peak accounted for 8.9 ± 1.3% (*n* = 3) of the radioactive signal, whilst in TS *Apoe*^–/–^ mice, this peak accounted for 17.5 ± 4.5% (*n* = 4, *P* = 0.0286) of the signal (Fig. 3d), suggesting enhanced metabolism or reactivity of [^68^Ga]Ga-IEMA in TS compared with non-TS *Apoe*^–/–^ mice. These results show that in vivo [^68^Ga]Ga-IEMA has favorable blood kinetics (half-lives fast = 3.1 min and slow = 37.8 min) with mostly renal clearance.

### Enhanced retention of [^68^Ga]Ga-IEMA in unstable murine atherosclerotic plaque

**Fig. 4:**
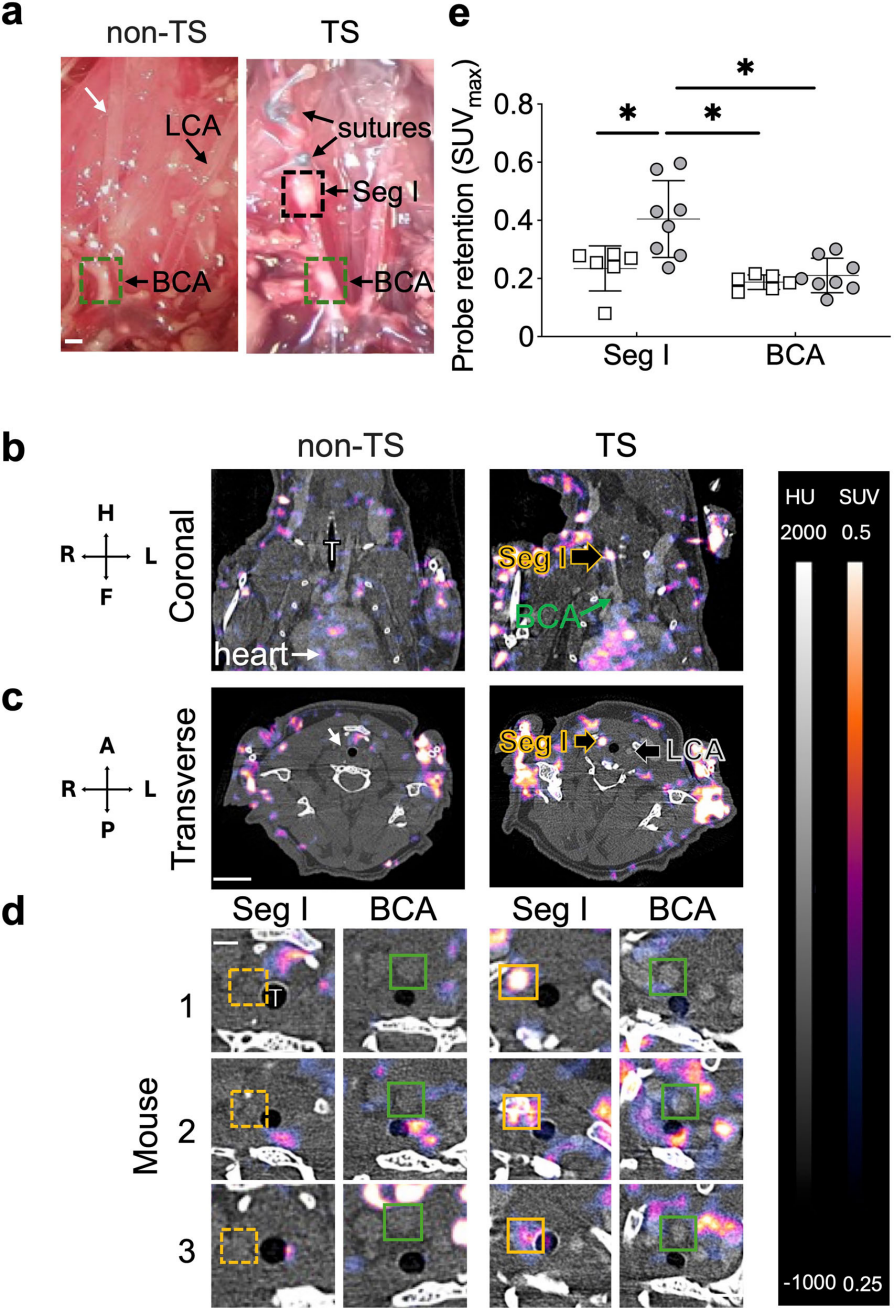
Retention of [^68^Ga]Ga-IEMA in stable and unstable atherosclerotic plaques in Apoe^–/–^ mice. **a** Representative in situ images after dissection show (i) the presence of atherosclerotic lesions in the brachiocephalic artery (BCA) known to contain stable lesions in both Apoe^–/–^ mice without (non-TS) and with Tandem Stenosis (TS); (ii) the selective presence of atherosclerotic lesions known to have characteristics of unstable plaque in Segment I (Seg I) of TS but not the corresponding lesion-free anatomical location in non-TS mice (white arrow); and (iii) the lesion-free left carotid artery (LCA) (scale bar = 0.5 mm). **b** Representative whole-body coronal images of in vivo PET imaging with [^68^Ga]Ga-IEMA in non-TS (*n* = 6) and TS Apoe^–/–^ mice (*n* = 8) 50–60 min after administration of the radiotracer (102.6 ± 1.5 µL, 8.2 ± 3.8 MBq). **c** Transverse images at the level of Seg I and contralateral LCA show increased retention of [^68^Ga]Ga-IEMA in Seg I compared with the anatomical location corresponding to Seg I in control non-TS Apoe^–/–^ mice, where plaque is absent (white arrow) (scale bar = 2 cm). **d** Zoomed images taken at the level of Seg I and the BCA from three different mice per group showing increased signal due to increased retention of [^68^Ga]Ga-IEMA in Seg I (yellow box) compared with the BCA in TS Apoe^–/–^ mice (green box). The images also confirm a lack of retention [^68^Ga]Ga-IEMA in the site corresponding to Seg I in non-TS Apoe^–/–^ mice (broken yellow box) (scale bar = 0.5 mm). **e** Maximum standardized uptake value (SUV_max_) of Seg I and BCA obtained from in vivo PET images 50–60 min post-injection of [^68^Ga]Ga-IEMA in non-TS Apoe^–/–^ (open squares) and TS Apoe^–/–^ mice (filled circles). *P < 0.05 (ANOVA with a Tukey post hoc test). T trachea.

After characterization of the [^68^Ga]Ga-IEMA in vitro and in vivo, we investigated the utility of the new radiotracer to non-invasively detect elevated MPO activity known to be present in Segment I of TS *Apoe*^–/–^ mice characterized by the presence of unstable atherosclerotic plaque^25,37^ (Fig. 4a). Representative in situ images after dissection show the development of atherosclerosis in the BCA in both non-TS and TS *Apoe*^–/–^ mice and the formation of unstable plaque selectively in Segment I of TS but not non-TS mice (Fig. 4a). Out of the ten mice undergoing TS surgery, eight developed unstable plaques in Segment I based on visual assessment and judged by the presence of both, positive remodeling and hemorrhage, at the time animals were euthanized. Two animals did not develop unstable plaques and were excluded from the analysis. In vivo coronal and transverse PET/CT images acquired from non-TS *Apoe*^–/–^ and TS *Apoe*^–/–^ mice identified the heart and the vasculature extending from the aortic arch, Segment I, and the contralateral, lesion-free left carotid artery (Fig. 4b, c). Transverse zoomed images acquired from six out of eight TS *Apoe*^*–/–*^ animals showed a visually increased PET signal due to retention of [^68^Ga]Ga-IEMA in unstable plaque-containing Segment I compared with that observed at the BCA (Fig. 4d), where stable plaques are formed. Similarly, retention of [^68^Ga]Ga-IEMA in unstable plaque-containing Segment I of TS *Apoe*^–/–^ mice was enhanced when compared with that observed at that same anatomical location in six non-TS *Apoe*^–/–^ mice (Fig. 4d) where plaque is absent (Fig. 4a). Quantification of the molecular PET signal using the maximum standardized uptake value (SUV_max_) showed significantly higher retention of [^68^Ga]Ga-IEMA in Segment I (0.4 ± 0.13, mean ± SD) than the BCA (0.2 ± 0.06) (*P* < 0.05) of TS *Apoe*^–/–^ mice (*n* = 8) and the same anatomical locations as Segment I (0.2 ± 0.07) and BCA (0.19 ± 0.02) in non-TS *Apoe*^–/–^ mice (*n* = 6) (*P* < 0.05).

### Enhanced retention of ^nat^Ga-IEMA in ruptured/eroded rabbit atherosclerotic plaque

After demonstration that molecular PET using [^68^Ga]Ga-IEMA can identify unstable plaques in Segment I in the TS mouse model, we performed an ex vivo proof-of-principle study to investigate whether the newly developed probe becomes retained in ruptured or eroded plaques in a rabbit model of atherothrombosis (Fig. S1). Out of 10 rabbits, 7 developed atherosclerosis in the aorta, and 4 advanced to trigger-induced atherothrombosis. MR angiograms (MRA) show the abdominal and thoracic aorta (Fig. S2a). T1BB images without contrast enhancement acquired before triggering show the presence of atherosclerotic plaque and the formation of additional luminal narrowing because of thrombosis (Fig. S2b). Determination of ^nat^Ga showed a significantly higher retention of the probe in thrombosed compared with stable plaques in this model (17.5 ± 3.9 vs 8.5 ± 1.6 ng/g, *P* < 0.05) (Fig. S2c). Together, these results show that in vivo imaging with [^68^Ga]Ga-IEMA PET/CT selectively identified unstable plaque in the mouse model of plaque instability. Consistently, [^nat^Ga]Ga-IEMA retention was higher in ruptured/eroded than in stable plaques in rabbits. Therefore, [^68^Ga]Ga-IEMA enables non-invasive PET imaging of extracellular MPO activity and demonstrates robust performance across small and large animal models.

## Discussion

We sought to develop an activatable radiotracer, [^68^Ga]Ga-IEMA, for in vivo molecular PET imaging of extracellular MPO activity for the detection of high-risk atherosclerotic plaque. Our data show, for the first time, that non-invasive molecular PET/CT imaging of extracellular MPO activity with [^68^Ga]Ga-IEMA selectively identifies and distinguishes unstable from stable plaque in vivo. These findings highlight the translational potential of [^68^Ga]Ga-IEMA PET/CT imaging to improve the identification of high-risk atherosclerotic disease based on assessing disease activity.

The results show that molecular PET imaging using the [^68^Ga]Ga-IEMA radiotracer provides an index of extracellular MPO activity enabling the non-invasive detection of unstable atherosclerotic plaque. This conclusion is supported by several lines of evidence. First, in vitro chemical characterization showed that the radiosynthesis of [^68^Ga]Ga-IEMA is quick and effective with high radiochemical yield, and that [^68^Ga]Ga-IEMA is moderately hydrophilic and, unless “activated”, is stable in human serum. Second, the new radiotracer requires the presence of enzymatically active MPO to oligomerize and bind to albumin, but does not cross cell membranes. This allows the probe to predominantly accumulate in the extracellular spaces of sites with elevated MPO activity. Third, the [^68^Ga]Ga-IEMA radiotracer remains stable in circulation and has favorable in vivo pharmacokinetics characterized by a bi-phasic blood clearance with predominant (~73%) secretion via the urine, and minor elimination via by the liver/bile (~17%) and intestine (~8%) as measured by ex vivo gamma counting at 90 min post-injection. Lastly, the radiotracer is retained specifically in unstable plaque that is characterized by elevated MPO protein and activity^25,26^. This resulted in significantly higher molecular PET signal compared with stable plaque and plaque-free segments. The results of the present study agree with previous findings in animal and human models showing that molecular imaging of extracellular MPO activity with gadolinium-based MRI contrast agents can selectively enhance histologically unstable plaque and monitor treatment response in the mouse model of plaque instability^25,26,31^ and can detect inflamed^34^ and thrombosis-probe atherosclerotic plaques in rabbits^27^. The results presented in this study were obtained with the newly developed radiotracer, [^68^Ga]Ga-IEMA, that is more suitable for clinical translation due to lower regulatory requirements compared with molecular MRI contrast agents.

There were two key considerations when designing the new radiotracer [^68^Ga]Ga-IEMA. Firstly, and like the MPO-activatable MRI probe heMAMP^31^, we employed two 5-hydroxyindole moieties as “MPO-activatable” units. This is because small molecular weight compounds containing 5-hydroxyindole are substrates of MPO, and the reduction potential of 5-hydroxyindole is such that it readily undergoes a 1-electron oxidation reaction with both intermediates of active MPO, i.e., compounds I and II, thereby forming a 5-hydroxyindole-derived radical and recycling native MPO^38^. The presence of 5-hydroxyindole moieties also provides specificity for MPO, as other mammalian peroxidases (eosinophil peroxidase, lactoperoxidase) do not effectively oxidize or activate 5-hydroxyindole-containing probes^38^. Moreover, the coupling to two moieties of 5-hydroxyindoles via two amide bonds (Fig. 1) increases binding to the active site of MPO, thereby increasing the ability of the probe to be activated by MPO^31^. Together, these features provide [^68^Ga]Ga-IEMA with high specificity and sensitivity to react with enzymatically active MPO. Our findings confirm that [^68^Ga]Ga-IEMA was activated readily by MPO and subsequently formed oligomers and bound to albumin similarly to other MPO-activable MRI and PET probes^30,38^. Although we did not investigate the structures of the oligomers in our study, previous work using analogous MPO-activated imaging probes showed that such oligomers are composed of oxidatively coupled probe molecules and probe–protein conjugates formed via radical chemistry initially involving one-electron transfer of the 5-hydroxytryptamide moieties of the probe with compound I in the catalytic cycle of MPO to form radicals that escape MPO’s active site and engage in radical-radical coupling to give rise to dimers, higher-order oligomers, and protein-anchored networks that increase relaxivity (for MPO MRI probes) and tissue retention^31,38^.

Secondly, we focused on the overall molecular charge of the new radiotracer. In contrast to the MPO-activatable MRI probe (heMAMP)^31^ that contains DOTA, we chose NODAGA as the chelator. NODAGA has one less pendant carboxylate arm than DOTA, resulting in an overall neutrally charged radiotracer when complexed with Ga^3+^ compared with a negatively charged Gd-DOTA complex^31^. A charged probe is expected to be excreted more rapidly compared with a neutral probe. Indeed, our in vivo pharmacokinetics data confirmed that the fast- and slow-phase blood half-lives of [^68^Ga]Ga-IEMA are 3.5 and 41.2 min in non-TS and 3.1 and 37.8 min in TS *Apoe*^*–/–*^ mice (Fig. 3) compared with 1.2 and 31.7 min, respectively, reported for heMAMP^31^. Considering the significantly lower injected dose used for a PET radiotracer (nano–micromolar) compared with a gadolinium-based MRI agent (millimolar), the longer slow-phase half-life of [^68^Ga]Ga-IEMA is advantageous, allowing enough time for the probe to reach its target in amounts sufficient to elicit a molecular PET signal. Importantly, the observed half-lives for [^68^Ga]Ga-IEMA are even more favorable when compared with those reported for the MPO-activatable radiotracer ^18^F-MAPP, i.e., 0.26 and 4.66 min, respectively^32^. Finally, characterization of the metabolism of the radiotracer in vivo showed a “secondary peak” in urine with shorter elution time that was greater in TS than non-TS mice, suggesting conversion of [^68^Ga]Ga-IEMA to a more polar, likely oxidized derivative. Separate experiments revealed that the elution time of this “secondary peak” is different to those of probe oligomers and the probe-albumin adduct. We considered chemical characterization of the “secondary peak” to be outside the scope of the present manuscript. Previous studies by others reported that plaque instability associates with increased circulating concentrations of the microbiome-derived metabolite trimethylamine-N-oxide, itself produced via oxidation of trimethylamine by flavin-containing monooxygenases in the liver^39^.

An additional advantage of using NODAGA over DOTA as a chelator is that radiolabelling the non-radioactive precursor with ^68^Ga can be achieved under mild conditions and with low precursor concentrations, leading to higher molar activities and reducing the likelihood of target saturation by the non-radioactive precursor. The simplicity of NODAGA radiochemistry, requiring a single 10-min reaction step without a need for further purification, also compares favorably with the two-step radiosynthesis of the ^18^F-MAPP MPO-activable radiotracer^32^. We conducted all radiolabelling at 50 °C for consistency, although separate experiments showed that radiolabelling using the same protocol at room temperature was also successful. By comparison, radiosynthesis of ^18^F-MAPP^32^ required heating to 70 °C, a reaction time of 17 min plus an additional purification step, with an overall activity yield less than the 89% achieved for the single-step radiosynthesis of [^68^Ga]Ga-IEMA.

In contrast to the cell-permeable ^18^F-MAPP (logP = 0.41)^32^, [^68^Ga]Ga-IEMA had a moderate to high hydrophilicity (logP = –2.23 to –2.30) as determined by octanol-water partitioning experiments. Indeed, in vitro experiments confirmed that [^68^Ga]Ga-IEMA does not cross cell membranes, as predicted by Lipinski’s Rule of 5^35,36^, and consistent with other probes of comparable hydrophilicity reported to have favorable pharmacokinetics for in vivo imaging of the vasculature^40,41^. Therefore, [^68^Ga]Ga-IEMA primarily detects deleterious extracellular MPO activity.

In vivo PET imaging of atherosclerotic plaques in mice presented several challenges. First, locating the vasculature, and specifically the carotid and brachiocephalic arteries, necessitated the use of a gold nanoparticle-based blood-pool contrast agent and acquisition of high-resolution/zoomed CT images to achieve the necessary spatial resolution. Second, the high positron emission energy of gallium-68 (*E*_max_ = 1.899 MeV) combined with the small size of the mouse arteries required optimization of the parameters to reconstruct the in vivo PET images to overcome partial volume effects. After overcoming these challenges, we demonstrated that PET/CT imaging with [^68^Ga]Ga-IEMA is sufficiently sensitive to detect increased probe retention in unstable plaque in TS *Apoe*^–/–^ mice. Indeed, in vivo PET/CT revealed consistent, specific, and enhanced retention of [^68^Ga]Ga-IEMA in Segment I that contains unstable plaque, resulting in a strong molecular PET signal. Conversely, the brachiocephalic artery, where stable plaques are present in TS *Apoe*^–/–^ mice^25,37^, and plaque-free arterial segments showed significantly less PET signal. Previous MPO imaging experiments in *Mpo*^*−/−*^*Apoe*^*−/−*^ mice using an MRI probe analogous to the PET radiotracer developed in this study, showed that genetic deletion of MPO (or pharmacological inhibition of MPO) stabilized plaques in the TS model and led to reduction of the MPO-MRI signal in the right carotid artery, *i.e*., the region where unstable plaque form, confirming that activation of this tracer is MPO-dependent^25,26^. Also, the imaging findings in previous studies were validated by biochemical and histological analyses. Moreover, previous studies demonstrated the specificity of 2-hydroxytryptamide-based probes for MPO compared with other peroxidases, based on the standard reduction potential relative to the MPO intermediates, i.e., 1.35 V for compound I/compound II, and 0.97 V for compound II/native state^38^.

Considering that the TS mouse model of plaque instability does not routinely result in plaque rupture and thrombosis, we used a rabbit model to assess the retention of the newly developed probe in plaques that advance to atherothrombosis. In this model, plaque disruption is pharmacologically “triggered” and depends on an unstable plaque phenotype, and is caused by both plaque rupture and erosion^42,43^, thereby resembling atherothrombosis in humans^44,45^. Limited by the lack of access to a PET/CT scanner that can accommodate rabbits, we used an ex vivo proof-of-principle approach to show higher retention of “cold” ^nat^Ga-IEMA in ruptured/eroded compared with stable plaques. These findings are in agreement with previous work in this rabbit model showing that in vivo molecular MRI using a Gd-MPO probe can prospectively and non-invasively identify thrombosis-probe atherosclerotic plaques with high sensitivity and specificity based on elevated MPO activity as verified separately using a highly specific LC-MSMS method^27^.

There has been continued interest in establishing PET imaging to non-invasively image arterial inflammation in relation to high-risk atherosclerotic plaque, with most emphasis focusing on the detection of macrophages. The PET radiotracers [^18^F]-FDG and [^68^Ga]Ga-DOTATATE detect macrophages via non-specific enhanced glucose uptake^12^ and the expression of the somatostatin receptor subtype-2, respectively^46,47^. However, [^18^F]-FDG imaging is susceptible to changes in blood glucose concentrations^48^, generates signals with high heterogeneity^49^, and is subject to interference due to high glucose uptake by cardiomyocytes. The somatostatin receptor subtype-2 is expressed on various cells, including pro- and anti-inflammatory macrophages, and its immune regulatory function remains to be established^46,47^. Importantly, given their lack of specificity for pro-inflammatory macrophages, [^18^F]-FDG and [^68^Ga]Ga-DOTATATE do not provide a direct measure of disease activity causing plaque instability. As such, a clinically available imaging agent that specifically and directly measures disease activity causing plaque destabilization remains elusive.

We show that molecular PET/CT with [^68^Ga]Ga-IEMA can non-invasively and selectively identify and differentiate unstable from stable plaque based on its ability to detect extracellular MPO activity. We posit that [^68^Ga]Ga-IEMA is a realistic candidate for translational PET/CT angiographic imaging to aid clinical detection of vulnerable plaques in coronary and carotid arteries. The dose of [^68^Ga]Ga-IEMA required is extremely low (≤50 μg per human subject), and several [^68^Ga]Ga-NODAGA-based probes have successfully been used in first-in-human studies over the past years worldwide^50^. This has been facilitated by the fact that ^68^Ga radiochemistry only requires a ^68^Ge/^68^Ga generator rather than a cyclotron, the radiochemistry is simple/amenable to kit formulation, [^68^Ga]Ga-NODAGA complexes have high thermodynamic stability, and their short half-life results in low radiation exposure to the subject. Finally, our dosimetry data for [^68^Ga]Ga-IEMA indicated comparable radiation exposure to that of clinically used ^68^Ga radiotracers, including [^68^Ga]Ga-PSMA (4.6 mSv/200 MBq), and [^68^Ga]Ga-DOTATATE (6.4 mSv/250 MBq), as well as [^18^F]-FDG (3.8 mSv/200 MBq)^51^. This further facilitates and supports clinical translation of [^68^Ga]Ga-IEMA.

Although no significant difference was observed between the pharmacokinetics of [^68^Ga]Ga-IEMA in non-TS *Apoe*^*–/–*^ and TS *Apoe*^*–/–*^ mice, an increased amount of activated probe was present in the urine of TS *Apoe*^*–/–*^ mice 90 min after injection of [^68^Ga]Ga-IEMA. We did not attempt to identify the reason for this, although a possible explanation is the heightened level of systemic oxidative stress associated with TS surgery. Although the TS mouse model develops histologically unstable plaques, it does not routinely develop plaque rupture or erosion and associated thrombosis. Therefore, using the TS mouse model, we could not test whether molecular PET using [^68^Ga]Ga-IEMA enables discrimination of plaques that rupture or erode from those that do not in vivo. As we did not have access to a PET/CT scanner that can accommodate rabbits, the experiments performed in the rabbit model of atherothrombosis used cold ^nat^Ga-IEMA and ex vivo elemental analysis of plaques by ICP-MS. Future PET experiments are needed to test the sensitivity and specificity of [^68^Ga]Ga-IEMA to prospectively identify thrombosis-probe plaque in vivo.

We did not study the kinetics of the enzymatic transformation of [^68^Ga]Ga-IEMA. However, previous studies have studied the pseudo-first-order rate constant for the enzymatic transformation of the related MRI MPO-specific probe [5-hydroxytryptamin-DOTA(Gd)] in the presence of MPO and H_2_O_2_ in vitro^30^. These studies showed fast kinetics with a pseudo-first-order rate constant of 6.3 × 10^−3 ^s^−1^ and >80% of the final change obtained within 5 min. The same study added 5-hydroxytryptamin-DOTA(Gd) to MPO-containing Matrigel matrix to test probe activation in a tissue model system and reported MRI signal changes after 1 h of incubation. In line with that study^30^, we kept the incubation period to 1 h for the in vitro characterization of [^68^Ga]Ga-IEMA. Based on the in vivo PET/CT imaging, we observed significant retention of [^68^Ga]Ga-IEMA and elevated PET signal 50–60 min after administration of the probe. These results suggest slow in vivo transformation of [^68^Ga]Ga-IEMA is not a limiting factor. Finally, this study did not test whether the newly developed radiotracer can be used to monitor treatment response to MPO inhibition.

Our data show the utility of a new PET radiotracer to provide an index of extracellular MPO activity as a non-invasive and clinically translatable method to identify high-risk atherosclerotic plaque. This study suggests that translation of this approach to humans may enable the identification and evaluation of therapies targeting arterial inflammation in patients at risk of developing acute cardiovascular events in a precision medicine approach. Future first-in-human safety trials are warranted.

## Methods

Unless indicated otherwise, chemicals used for the synthesis of the PET radiotracer, [^68^Ga]Ga-IEMA (Index of Extracellular Myeloperoxidase Activity) were purchased from Sigma-Aldrich, Acros Organics/Thermo Fisher Scientific, or Fluorochem UK.

### Analytical methods

Analytical high-performance liquid chromatography (HPLC) was carried out using an Agilent 1260 Infinity II system with a diode array or a multi-wavelength detector. Semi-preparative HPLC data were acquired on an Agilent 1260 Infinity II system featuring a preparative binary pump and a multi-wavelength detector. Data was processed and analyzed using OpenLab CDS software (Agilent Technologies, USA). Radio-HPLC and radio-ITLC data were acquired using a plastic PMT detector interfaced with a Dual Scan-Ram (LabLogic Systems, UK) with data analyzed using Laura 6.2 software (LabLogic Systems, UK). HPLC methods are described in Table S1. Nuclear Magnetic Resonance (NMR) data were acquired on a Bruker 400 MHz and analyzed using MestReNova 14.2 software (Mestrelab Research S.L., Spain). Mass spectrometry (MS) data were acquired on an Advion Expression^L^ compact mass spectrometer using an electrospray ionization source in positive polarity, capillary and source gas temperature of 250 °C, capillary voltage of 120 V, source voltage offset of 20 V, and a source voltage span of 40 V.

### Synthesis of PET radiotracer, [^68^Ga]Ga-IEMA

*Synthesis of intermediate 1—*Di-*tert*-butyl dicarbonate (1.19 g, 5.5 mmol) dissolved in tetrahydrofuran (THF, 6 mL) was added to a solution of 5-hydroxy-L-tryptophan (1.00 g, 4.5 mmol) in aqueous potassium carbonate (2.08 g, 15.1 mmol in 10 mL water) and stirred at room temperature (RT) for 2 h. Hydrochloric acid (1 M) was added until pH 2–3 was reached, and the THF evaporated under reduced pressure. The aqueous phase was then extracted with ethyl acetate (3 × 30 mL), before the organic phase was washed with brine (3 × 20 mL), dried over anhydrous sodium sulfate, and evaporated under reduced pressure to yield a sticky yellow-brown solid (intermediate 1) that was used without further purification.

*Synthesis of intermediate 2—*Intermediate 1 (385 mg, 1.2 mmol), 1-[*bis*(dimethylamino)methylene]-1*H*-1,2,3-triazolo[4,5-*b*]pyridinium 3-oxid hexafluorophosphate (HATU, 763 mg, 2.0 mmol) and diisopropylethylamine (475 µL, 352.5 mg, 2.72 mmol) were dissolved in dry dimethylformamide (DMF, 10 mL) and stirred at RT under argon for 30 min. 5-Hydroxy-L-tryptophan hydrochloride (220 mg, 1.0 mmol) in dry DMF (5 mL) was added, and the reaction mixture was stirred at RT under argon overnight. Ethyl acetate (100 mL) was then added, and the reaction mixture was washed with water (3 × 50 mL). The organic phase was then washed with brine (3 × 50 mL), dried over anhydrous sodium sulfate, and evaporated under reduced pressure. The crude product was dry-loaded onto a 120 g Biotage® Sfär C18 Duo column using celite 545, and compounds separated by reversed-phase flash HPLC using a gradient ranging from 95% solvent A (0.1%, vol/vol, formic acid in water)/5% solvent B (0.1% formic acid in acetonitrile) to 100% solvent B (Method 1). The product, eluting as a single peak at ~35% solvent B, was identified by electrospray ionization-mass spectrometry (ESI-MS) based on its molecular weight of [M + H]^+^ *m/z* = 479.2; calc. for C_26_H_31_N_4_O_5_ = 479.2. The fractions containing the product were combined and lyophilized to yield a white powder (intermediate 2, 147 mg, 307 µmol, 31% yield after 2 steps). ^1^H NMR (400 MHz, DMSO-d_6_) δ 10.46 (s, 2H), 8.58 (s, 1H), 8.55 (s, 1H), 7.90 (t, 1H), 7.11 (d, 1H), 7.10 (d, 1H), 7.00 (m, 2H), 6.89 (d, 1H), 6.83 (d, 1H), 6.68 (d, 1H), 6.59 (dd, 2H), 4.14 (m, 1H) 3.28 (m, 2H), 2.97 (dd, 1H), 2.80 (dd, 1H), 2.67 (m, 2H), 1.33 (s, 9H).

*Synthesis of intermediate 3—*Intermediate 2 (143 mg, 3.0 mmol) was dissolved in dichloromethane/trifluoroacetic acid (10:1 vol/vol, 4 mL) and stirred at RT overnight, before the solvent was removed under reduced pressure. The crude mixture was loaded as an aqueous solution onto a 12 g Biotage® Sfär C18 Duo column, and compounds separated by reversed-phase flash HPLC using the gradient described above for intermediate 2. The product, eluting at ~6% solvent B, was collected and lyophilized to yield a white powder (intermediate 3, 17.7 mg, 46.8 µmol, 16%). ^1^H NMR (400 MHz, DMSO-d_6_) δ 10.64 (d, 1H), 10.47 (d, 1H), 8.58 (m, 2H). 8.38 (t, 1H), 7.12 (dd, 2H), 7.06 (d, 1H), 6.96 (dd, 2H), 6.80 (d, 1H), 6.62 (dd, 1H), 6.58 (dd, 1H), 3.74 (dd, 2H), 3.35 (m, 2H), 3.06 (dd, 1H), 2.88 (dd, 1H), 2.66 (m, 2H). ESI-MS: [M + H]^+^ *m/z* = 379.2; calculated for C_21_H_23_N_4_O_3_ = 379.2.

*Synthesis of IEMA—*Intermediate 3 (4.02 mg, 10.6 µmol) was mixed with NODAGA-NHS ester (21.0 mg, 46.0 µmol) in dry DMF (500 µL). Dry diisopropylethylamine (10 µL, 7.42 mg, 57.4 µmol) was then added, and the reaction mixture was stirred at RT under argon overnight. DMF was removed under a vortex of air at 40 °C. The crude product was purified by semi-preparative HPLC (Method 2) and lyophilized to yield a white powder (*IEMA*, 3.63 mg, 4.93 µmol, 47%). ^1^H NMR (400 MHz, DMSO-d_6_) δ 10.53 (d, 1H), 10.50 (d, 1H), 8.66 (br s, 2H), 8.18 (m, 1H), 8.15 (m, 1H), 7.15 (d, 1H), 7.11 (d, 1H), 7.08 (d, 1H), 7.03 (dd, 2H), 6.85 (d, 1H), 6.56 (m, 2H), 4.40 (m, 1H), 3.62 (s, 4H), 3.25 (br m, 4H), 2.95 (br m, 10H), 2.77 (m, 2H), 2.70 (t, 2H), 2.37 (m, 1H), 2.16 (m, 1H), 1.96 (m, 1H), 1.69 (m, 1H). [M + H]^+^ *m/z* = 736.9; calculated for C_36_H_46_N_7_O_10_ = 736.8.

### Radiosynthesis of [^68^Ga]Ga-IEMA

Formate buffer (1.2 M) was prepared by adding formic acid (492 µL, 13 mmol) to an aqueous solution of sodium hydroxide (6 mL, 2.5 M), followed by adjusting the volume to 10 mL by the addition of water (3.508 mL). [^68^Ga]GaCl_3_ was eluted from a ^68^Ge/^68^Ga generator (Eckert & Ziegler) in hydrochloric acid (5 mL, 0.1 M, 250–500 MBq) in sequential fractions of 1.5, 1.0, 1.0, and 1.5 mL. The second fraction, containing 75–80% of the total radioactivity, was retained, and the other fractions discarded. Aqueous IEMA (10 µL, 1 mg mL^–1^, 1.36 mM) and sodium formate buffer (40 µL) were added to a reaction vessel, followed quickly by [^68^Ga]GaCl_3_ (400 µL, 180–450 MBq) before the solution (pH ~3.5) was mixed briefly. The reaction mixture was heated to 50 °C for 10 min before being neutralized to pH 7.0–7.5 by the addition of aqueous sodium bicarbonate (35 µL, 1 M). Within 1 min of completion of the reaction, the reaction mixture was subjected to instant thin-layer chromatography (Agilent Technologies glass microfibre chromatography paper impregnated with silica gel) using a mobile phase of methanol/1.3 M aqueous ammonium acetate (1:1 vol/vol). Radiochemical yield and purity of separated compounds (unbound ^68^Ga *R*_f_ = 0–0.2; [^68^Ga]Ga-IEMA *R*_f_ = 0.7–1.0) were evaluated, and a 95% radiochemical purity was used as a threshold for quality control in all experiments. In addition, purity of [^68^Ga]Ga-IEMA was assessed by radio-reversed-phase HPLC (Method 3, *t*_R_ = 10.82 min) within 2–3 min of the end of the reaction.

### In vitro characterization of [^68^Ga]Ga-IEMA

*LogP and LogD*_*7.4*_*—*[^68^Ga]Ga-IEMA (10 µL, 2.6–2.8 MBq), formulated in a solution as described in the Radiosynthesis section, was added to octanol pre-saturated with water (500 µL) and water pre-saturated with octanol (500 µL). The mixture was shaken vigorously at 37 °C for 30 min, centrifuged at 16,060 × *g* for 3 min, before 50 µL aliquots of the aqueous and organic layers were taken for radioactivity determination using a gamma counter (1282 CompuGamma, LKB-Wallac, Finland). LogD_7.4_ was determined by the same method using octanol pre-saturated with phosphate-buffered saline, pH 7.4 (PBS), and PBS pre-saturated with octanol in place of their water counterparts.

*Stability of [*^*68*^*Ga]Ga-IEMA in serum—*[^68^Ga]Ga-IEMA (50 µL, 7.5–8.9 MBq), formulated in a solution as described in the Radiosynthesis section, was added to a mixture of freshly filtered human serum (250 µL, H4522-20ML, Merck Life Science, USA) and PBS containing 0.5% sodium ascorbate (vol/wt, 250 µL) and incubated at 37 °C. Aliquots (50 µL) were removed after 1, 2, and 3 h and analyzed by size-exclusion radio-HPLC (HPLC Method 4).

*Protein binding in solution—*[^68^Ga]Ga-IEMA (100 µL, 28–30 MBq), formulated in a solution as described in the Radiosynthesis section, was incubated in 150 µL PBS containing 1 μL human MPO (5 mg/mL, ab77886, Abcam, UK), 5 μL glucose oxidase (GOX, 0.2 mg/mL, G2133, Sigma, USA), 40 µL D-(+)-glucose (250 mg/mL, G8270, Sigma, USA) and 12 mg bovine serum albumin (BSA) for 60 min at 37 °C with stirring at 1000 rpm, before the mixture was analyzed by size-exclusion radio-HPLC (Method 5).

*Gel retention study—*Human MPO (1 µL, 5 mg/mL, ab77886, Abcam, UK) was dissolved and embedded in a hybrid gel prepared from a mixture of 18.5% acrylamide (30% acrylamide/bis solution, 1610158, Bio-Rad, USA) and 5% Matrigel (22 mg protein/mL, 356234, Corning, USA). MPO-free control gels were prepared by substituting the MPO-containing solution with an equal volume of PBS. For studying retention, the gel was incubated with [^68^Ga]Ga-IEMA (20 µL, 0.5–0.6 MBq) for 1 h at 37 °C with shaking in 200 µL PBS (no MPO activation) or 180 µL PBS to which was added 10 µL GOX (40 µg/mL) and 10 µL D-(+)-glucose (20 mg/mL) each dissolved in PBS. Following incubation, the supernate was collected and the gel washed with PBS (3 × 200 µL, pH 7.4) prior to transfer into a clean tube. The radioactivity of each gel and its corresponding supernate and washes was determined using a gamma counter (1282 CompuGamma). The percentage of [^68^Ga]Ga-IEMA retained in the gel was calculated using the following equation: % Probe in gel = CPM_gel_/(CPM_gel_ + CPM_supernate_ + CPM_washes_) * 100. CPM: counts per minute.

*Cellular uptake—*Human umbilical vein endothelial cells (HUVEC, HEC01, Neuromics, USA) were used to confirm the lack of cellular uptake of [^68^Ga]Ga-IEMA. Cells were seeded in 6-well plates at a density of 2 × 10^5^ cells/well and cultured at 37 °C with 5% CO_2_ in fresh Endothelial Cell Growth Medium (EGM2; PromoCell, C-22011) for 20 h to allow sufficient cell attachment. Cells were then washed with PBS before DMEM-F12 containing 0.5% fetal bovine serum was added. [^68^Ga]Ga-IEMA (0.14 or 1.4 MBq) or free ^68^Ga (control, prepared in the same way as [^68^Ga]Ga-IEMA except that water was used in place of IEMA) was added directly to each well and mixed with the medium by gently pipetting before cells were incubated for 1 h at 37 °C. A single experiment was performed in triplicate for each condition. Following incubation, the supernates were collected, the cells washed 3 times with PBS, and the wash buffers collected. The cells were then detached by trypsin-EDTA (0.25%, Gibco™ 25200072, Thermo Fisher, Canada) treatment for 2 min at 37 °C, before trypsin was deactivated by adding DMEM-F12 containing 0.5% fetal bovine serum. Cell pellets and supernates were collected by centrifuging the samples at 400 × *g* for 5 min before determination of the radioactivity of all samples using a gamma counter. The following day, the protein content in cell lysates was determined using the Pierce^TM^ bicinchoninic acid protein assay kit as per the manufacturer’s instruction. Cellular uptake of [^68^Ga]Ga-IEMA was calculated using the following equation: Cellular uptake = CPM_cells_/(CPM_cells_ + CPM_supernates_ + CPM_washes_) and normalized to the cellular protein content. CPM: counts per minute.

### Animals

Male apolipoprotein E gene-deficient (*Apoe*^–/–^*)* mice purchased originally from Charles Rivers Laboratories (Edinburgh, UK) were housed and bred in the Biological Service Facility at King’s College London. All animal procedures were performed in accordance with the Animals (Scientific Procedures) Act 1986 and Amendment Regulations 2012, Guidance of Operations of the Animals (Scientific Procedures) Act 1986, and carried out under project license (PP8261525) granted from the United Kingdom of Great Britain and Northern Ireland Home Office. Animals were housed in accordance with the Code of Practice for the Housing and Care of Animals Bred, Supplied or Used for Scientific Purposes. Briefly, animals were maintained in groups of 2–4 or, in rare incidents of home-cage aggression, housed separately in the individually ventilated cages under controlled environmental conditions (12 h of light/dark cycle, at approximately 21 °C and humidity 55 ± 10%).

*Tandem stenosis (TS) mouse model of unstable atherosclerotic plaque—*Male *Apoe*^–/–^ mice (8 weeks of age, 33.5–44.8 g, *n* = 10) were fed Western Diet (WD) containing 22% fat and 0.15% cholesterol (824063, Special Diet Services, UK) for a total of 13 weeks *ad libitum*. Six weeks after commencement of WD, surgery to induce tandem stenosis (TS) was performed^25,37^. Briefly, mice were anesthetized with 2–4% isoflurane. An incision was made in the neck, and the right common carotid artery was exposed and dissected from surrounding connective tissues. Two stenoses with 150 µm outer diameter were placed with the distal stenosis 1 mm from the carotid bifurcation and the proximal stenosis 3 mm proximal from the distal stenosis. To control for the extent of constriction caused by the stenosis, a 150-μm needle (8-0, W1782, Ethicon, USA) was placed on top of the exposed right common carotid artery before a 6-0 blue-braided polyester fiber suture (3280-11, 0.7 Metric, TICRON, UK) was tied around both the artery and needle, and the needle then removed. In addition, blood flow was measured before and after the addition of each ligature using a perivascular flow module (TS420, Transonic, USA) and a 0.7 mm perivascular flow probe (MA0.7PSB, Transonic). Flow for each ligature in the TS was defined as 70% of baseline flow after addition of the distal ligature and 20% of baseline flow after addition of the proximal ligature. Post-surgery, buprenorphine (0.1 mg/kg animal weight, Buprecare, Animalcare, UK) was administrated intraperitoneally for pain management.

*Rabbit model of atherothrombosis and administration of the [*^*nat*^*Ga]Ga-IEMA—*Atherosclerosis was induced in 8-11-weeks-old male, New Zealand White rabbits (~2.5 kg, *n* = 10)^42^. Rabbits were fed with a chow diet supplemented with 1% (wt/wt) cholesterol for 8 weeks to induce hypercholesterolemia. Two weeks after commencement of the cholesterol diet, anesthesia was induced by intramuscular ketamine (Ketamidor, Chanelle Pharma, Ireland, 35 mg/kg) and xylazine (Rompun, Dechra, UK, 5 mg/kg) and maintained by inhalation of 2–4% isoflurane. While under general anesthesia, aortic endothelial denudation was induced using a nominally inflated 3 F Fogarty catheter (12TLW403F, Edward Lifesciences, USA) advanced through a 4 F sheath into the infrarenal aorta through a right femoral artery cutdown. Endothelial denudation was achieved by three sequential manual pullbacks over approximately 90–100 mm. The artery was then ligated, followed by muscular and skin closure using 3–0 Vicryl sutures. After 8 weeks of a cholesterol diet, rabbits were placed on normal chow for 4 weeks. At the end of the feeding period, atherothrombosis was induced by intraperitoneal administration of Russell’s Viper Venom (0.15 mg/kg, Enzyme Research, UK) followed by an intravenous injection of histamine (0.02 mg/kg, MP Biomedicals, USA) 30 min later. Two pharmacological triggers were performed 2 h apart. One hour after the second trigger, rabbits were injected with [^nat^Ga]Ga-IEMA (1 mL intravenous injection equivalent to 15 μg of radiotracer). Finally, 90 min after the administration of the radiotracer, rabbits were euthanized with an intravenous injection of 100 mg pentobarbitone/kg (Pentoject, 20% (wt/vol), Animalcare, UK). The abdominal aorta was collected and stored at −80 °C until further ex vivo analyses.

All animal procedures were performed in accordance with the guidelines of the United Kingdom Home Office Animal (Scientific Procedures) Act 1986, project license number PP8261525. Ethical approval was granted by King’s College London’s Animal Welfare and Ethical Review Body.

### In vivo PET/CT imaging of mice and ex vivo analyses

*PET/CT imaging—*These studies included 10 “TS *Apoe*^–/–^ mice” that received WD for 13 weeks with TS surgery performed at the end of week 6, and six control “Non-TS *Apoe*^–/–^ mice” that received WD for 13 weeks without TS surgery being performed. At the end of the intervention, mice were anesthetized by inhalation of isoflurane (1.5–3.5% in O_2_ at 1 L/min) and injected intravenously with gold nanoparticles as a CT contrast agent (AuroVist™ 15 nm; 100 µL, 200 mg Au/mL, 1115 A, Nanoprobes, Yaphank, USA). The following day, mice were anesthetized by inhalation of isoflurane (1.5–3.5% in oxygen) and the tail vein cannulated with a 28 G butterfly needle pre-flushed with heparinized saline. Each mouse was placed on a nanoScan® PET/CT scanner (Mediso, Budapest, Hungary) in a prone position, with anesthesia maintained. A whole-body CT (50 kV × 980 µA) was performed, followed by a high-resolution CT covering the upper chest and neck (35 kV × 980 µA). A 61- or 91-min PET scan was initiated, and after 60 s [^68^Ga]Ga-IEMA (102.6 ± 1.5 µL, 8.2 ± 3.8 MBq) was injected intravenously via the tail vein cannula. PET images were reconstructed using Tera-Tomo 3D reconstruction (400–600 keV energy window, 1–3 coincidence mode, 6 iterations and subsets) with a voxel size of (0.4 × 0.4 × 0.4) mm^3^ and correction for attenuation, scatter, positron range, and decay.

*Image analysis—*PET/CT imaging data were analyzed using InterView Fusion 3.11.008.0000 software (Mediso). Regions of interest (ROIs) were manually drawn using CT images to identify anatomical references including the left ventricle as a proxy for blood, the arterial segment near the proximal suture in the right common carotid artery (referred to as Segment I and known to contain plaque with an unstable phenotype^25,37^), and the brachiocephalic artery (BCA) known to contain plaques with stable features^25,37^. The use of gold nanoparticles, with high X-ray attenuation, enhanced image contrast, enabling clear delineation and segmentation of the vasculature of interest, i.e., Segment I and BCA. Signals from neighboring tissues were avoided. The mean standardized uptake value (SUV_mean_) was calculated to assess blood clearance. The maximum standardized uptake value (SUV_max_) was recorded for each ROI in Segment I and BCA.

Ex vivo *analyses of murine samples—*After imaging, mice were euthanized by intravenous administration of sodium pentobarbital (220 mg/kg). Urine was collected from the bladder in a syringe and analyzed for the presence of [^68^Ga]Ga-IEMA by reversed-phase HPLC (Method 3) without any further processing. Blood was collected via cardiac puncture, and then hearts were gently perfused with saline, and various tissues and organs were collected for ex vivo biodistribution measurements. Ex vivo biodistribution data are presented as SUV = (Activity_organ_/weight _organ_)/(Injected Activity/weight_mouse_).

In vivo *MRI of aortic plaques and thrombus in rabbits and* ex vivo *analyses—*In vivo MR images of the abdominal aorta were acquired using a 3.0 Tesla Philips Achieva clinical scanner (Philips Healthcare, Best, Netherlands) equipped with a clinical gradient system (30 mT/m, 200 mT/m per ms). Rabbits were anesthetized and positioned supine during the scan. Data acquisition was performed with a 32-channel cardiac coil and gated with a simulated electrocardiogram. At the end of the 12 weeks, rabbits were scanned twice, before triggering atherothrombosis (pre-trigger) and 1 h after the second trigger event using non-contrast enhanced T1-weighted black blood (T1BB) MRI. The pre-triggered MRI scan was used to identify plaques in the abdominal aorta, and the post-triggering scan was used to identify thrombosis resulting from plaque rupture or erosion.

Coronal phase-contrast angiograms were acquired for visualization of the aorta, renal branches, and iliac bifurcation with the following parameters: FOV = 300 × 150 × 15 mm, matrix = 200 × 100, resolution = 1.5 × 1.5 mm, slices = 15, slice thickness = 1 mm, TR/TE = 20/3 ms, flip angle = 15°, and phase-contrast velocity = 150 cm/s. The maximum intensity projection images with full vision of the abdominal aorta from the left renal branch to the iliac bifurcation were used to plan the subsequent images. Electrocardiographically (ECG) triggered transverse multi-slice 2D zoom (reduced FOV) double inversion recovery T1BB images were acquired with the following parameters: FOV = 70 × 19 × 100 mm, matrix = 280 × 69, resolution = 0.25 × 0.25 mm, slices = 20, slice thickness = 5 mm; TR = two heartbeats, TE = 11 ms, flip angle = 15 °, and BB inversion delay = 350 ms.

*Matching of MR images and histological sections—*The distances from the aortic renal branches and the iliac bifurcation were used as internal anatomic markers to match the in vivo MR images and aortic sections as reported^42^. During extraction, the abdominal aorta was marked with suture ligature at distances above and below the left renal branch that matched the total length covered by the in vivo MRI slices and was measured in situ prior to dissection. After extraction, the ligatures were used to stretch the aorta to its physiological length, marked at 1 cm intervals, and pinned to a corkboard. All samples were stored at −80 °C until further elemental analysis.

Ex vivo *inductively coupled plasma mass spectrometry (ICP-MS) analyses of rabbit samples*. Frozen rabbit aortic segments containing stable plaque (*n* = 13) and thrombosed plaque (*n* = 4) were selected from different rabbits based on MR images and gross views. Visible thrombi were removed from the tissue prior to analysis to quantify ^nat^Ga as a marker of the amount of [^nat^Ga]Ga-IEMA retained within the plaque. Samples were transferred to pre-weighed trace-grade centrifuge tubes (ElkayLabs, Hampshire, UK) and allowed to dry overnight in an oven at 70 °C. Samples were cooled and weighed before being digested by the addition of 0.5 mL Optima grade concentrated nitric acid (67–69% wt/wt; Fisher Scientific, Massachusetts, USA) and 0.25 mL hydrogen peroxide (30% w/w, Supelco, Pennsylvania, USA), followed by heating at 60 °C in a heat block for 1 h. Digested samples were then dissolved in 10 mL purified water with a resistivity of ≥18.2 MΩ cm (Milli-Q IQ 7015, Merck, Massachusetts, USA) and spiked with iridium (100 mg/L) as an internal standard at a final concentration of 10 µg/L. Resulting samples were analyzed at the London Metallomics Facility, King’s College London, selecting ^69^Ga for quantification of [^nat^Ga]Ga-IEMA and using ^193^Ir as internal standard on a NexION 5000 (Perkin Elmer, Massachusetts, USA) Inductively Coupled Plasma Mass Spectrometer (ICP-MS) under Dynamic Reaction Cell (Ammonia) mode coupled with a Cetac ASX-560 autosampler (Teledyne CETAC Technologies’, Nebraska, USA). Calibration standards were made volumetrically using 100 mg/L gallium standard solution, a 0.785 M nitric acid stock solution made from Optima grade concentrated nitric acid, and purified water. Element concentrations of the calibrants were between 0.1 and 100 µg/L. Data was collected using Syngistix version 3.5 (Perkin Elmer, Massachusetts, USA) and analyzed using a bespoke in-house pipeline, written in Python (v3.13) to normalize analyte measurements to the internal standard iridium to account for instrument drift and matrix effects. Measurements were subsequently blank-corrected by removing the average analyte intensity of repeat blank measurements. The corrected analyte intensity was converted to concentration using standard calibration curves. All standards were sourced from High-Purity Standards (South Carolina, USA).

### Dosimetry calculation

In vivo biodistribution data from mice were used to generate the predictive dose per organ in humans. The absorbed radiation dose for [^68^Ga]Ga-IEMA to various organs was calculated according to the Medical Internal Radiation Dose Committee of the Society of Nuclear Medicine using Olinda/EXM^52^.

### Statistical analysis

Statistical analysis was performed using GraphPad Prism 9.0 (GraphPad Software, Inc., La Jolla, California, USA). Normality was assessed using the Shapiro–Wilk test. For normally distributed data, a one-way analysis of variance (ANOVA) test followed by a Tukey post hoc test was used to compare across multiple groups. For non-normally distributed data, a Mann-Whitney test was performed for two group comparisons, while a Kruskal–Wallis ANOVA was used to compare across multiple groups. *P* < 0.05 was considered statistically significant. Data are presented as mean ± standard deviation (SD).

## Supplementary information

Supplemental Material-10.02.26-R1_QC_v2.

Supplementary Video S1-video-25.11.2025.

## Data Availability

The datasets generated and/or analyzed during the current study are not publicly available due to lack of access to repository but are available from the corresponding author on reasonable request.
