# Supplementary material for: Development and validation of an activatable PET radiotracer reporting extracellular myeloperoxidase activity for the detection of unstable atherosclerotic plaque
Source: Npj Imaging. 2026 Apr 7;4:23. doi: 10.1038/s44303-026-00156-9 (PMC13056917; doi:10.1038/s44303-026-00156-9)
Supplement: Supplementary file 1 — Supplemental Material-10.02.26-R1_QC_v2. [file 44303_2026_156_MOESM1_ESM.docx]

**Supplemental Material**

**Table S1**. HPLC methods used.

| Method | Column | Mobile Phase | Gradient | | | Flow rate (mL/min) |
| --- | --- | --- | --- | --- | --- | --- |
|  |  |  | Time  (min) | %A | %B |  |
| 1 | Agilent Eclipse XDB-C18 (4.6 × 150 mm, 5 μm) | Solvent A: 0.1% formic acid in water  Solvent B: 0.1% formic acid in acetonitrile | 0  2  11  12  12.1  15 | 95  95  5  5  95  95 | 5  5  95  95  5  5 | 1 |
| 2 | Agilent Eclipse XDB-C18 (9.4 × 250 mm, 5 μm) | Solvent A: 0.1% formic acid in water  Solvent B: 0.1% formic acid in acetonitrile | 0  5  50  51  60  61  70 | 95  95  80  5  5  95  95 | 5  5  20  95  95  5  5 | 5 |
| 3 | Agilent Eclipse XDB-C18 (4.6 × 150 mm, 5 μm) | Solvent A: 0.1% formic acid in water  Solvent B: 0.1% formic acid in acetonitrile | 0  5  20  25  25.1  30 | 95  95  5  5  95  95 | 5  5  95  95  5  5 | 1 |
| 4 | Phenomenex BioSep SEC-s2000 (7.8 × 300 mm, 5 µm, 145 Å) | PBS + 0.5% sodium ascorbate | 0–50 |  |  | 1 |
| 5 | Phenomenex BioSep SEC-s2000 (7.8 × 300 mm, 5 µm, 145 Å) | PBS | 0–35 |  |  | 1 |

**Figure S1.** **Enhanced retention of ^nat^Ga-IEMA in ruptured/eroded atherosclerotic plaque of rabbits. a.**MR angiogram. **b**. Pre-trigger T1BB images show aortic atherosclerotic plaques and thrombosis at arterial segments that advanced to trigger-induced atherothrombosis because of plaque rupture and/or erosion. **c.**Quantification of ^nat^Ga using ICP-MS shows significantly higher retention of ^nat^Ga-IEMA in ruptured/eroded plaques (open squares, n = 4) compared with stable plaques (open circles, n = 13) (17.5 ± 3.9 vs 8.5 ± 1.6 ng/g). P < 0.001 (Mann Whitney U). Ao: aorta, IVC: inferior vena cava.

**Movie S1.** Representative videos of the biodistribution of [^68^Ga]Ga-IEMA from 3 to 90 minutes post-injection showing both hepatic and renal clearance, as well as biliary excretion.
